# Transgenic tools for proteomic analysis of ciliary transport

**DOI:** 10.1186/2046-2530-4-S1-P43

**Published:** 2015-07-13

**Authors:** X Fang, U Jokopii, J Malicki

**Affiliations:** 1 University of Sheffield, Sheffield, UK; 2 University of Jyväskylä, Jyväskylä, Finland

## 

Vision begins as photons are captured by photoreceptor cilia and light is converted into electrical signals that are then sent to the brain. As the photoreceptor cilium is not able to make its own proteins, all polypeptides needed for converting photons into electrical signals are synthetized in the cell body. How these molecules move from the cell body to cilia is still unclear. Opsin is one of the best-characterized transmembrane proteins. Our goal is to understand the mechanism of opsin transport into photoreceptor cilia. In this project, we use a combination of genetic and proteomic approaches in the zebrafish model. As the first step, we are constructing a transgenic line that expresses EGFP-opsin fusion from an inducible promoter specifically in photoreceptors. For this purpose, we will mate two transgenic lines: 1. A line that specifically expresses Cre recombinase in photoreceptors, and 2. A line that conditionally expresses EGFP-opsinCT44 (EGFP fused with the 44 C-terminal residues of opsin, which are sufficient to mediate ciliary transport) from the heat-shock promoter. A lox-mCherry-STOP cassette is inserted upstream of EGFP-opsinCT44. In the long run, we plan to purify photoreceptors via FACS sorting, pull down EGFP-opsinCT44 using the EGFP tag, and analyze opsin C-terminal binding proteins by mass spectrometry. In parallel, we will perform analysis of opsin transport in cilia mutants. We hope this will allow us to formulate a general model of how transmembrane proteins, such as GPCRs and TRP channels, are transported into cilia.

